# A Molecular Survey of Bacterial Species in the Guts of Black Soldier Fly Larvae (*Hermetia illucens*) Reared on Two Urban Organic Waste Streams in Kenya

**DOI:** 10.3389/fmicb.2021.687103

**Published:** 2021-09-22

**Authors:** Marwa Shumo, Fathiya M. Khamis, Fidelis Levi Ombura, Chrysantus M. Tanga, Komi K. M. Fiaboe, Sevgan Subramanian, Sunday Ekesi, Oliver K. Schlüter, Arnold van Huis, Christian Borgemeister

**Affiliations:** ^1^Leibniz-Institute for Agricultural Engineering Potsdam-Bornim (ATB), Potsdam, Germany; ^2^Department of Ecology and Natural Resources Management, Center for Development Research (ZEF), Bonn, Germany; ^3^Plant Health Unit, International Centre of Insect Physiology and Ecology (ICIPE), Nairobi, Kenya; ^4^Hermetia Baruth GmbH, Insect Technology Center (ITC), Berlin, Germany; ^5^IPM Department, The International Institute of Tropical Agriculture, Yaoundé, Cameroon; ^6^Laboratory of Entomology, Wageningen University & Research, Wageningen, Netherlands

**Keywords:** organic waste treatment, black soldier fly larvae (BSFL), gut microbiota, insect rearing, feed safety, food security

## Abstract

Globally, the expansion of livestock and fisheries production is severely constrained due to the increasing costs and ecological footprint of feed constituents. The utilization of black soldier fly (BSF) as an alternative protein ingredient to fishmeal and soybean in animal feed has been widely documented. The black soldier fly larvae (BSFL) used are known to voraciously feed and grow in contaminated organic wastes. Thus, several concerns about their safety for inclusion into animal feed remain largely unaddressed. This study evaluated both culture-dependent sequence-based and 16S rDNA amplification analysis to isolate and identify bacterial species associated with BSFL fed on chicken manure (CM) and kitchen waste (KW). The bacteria species from the CM and KW were also isolated and investigated. Results from the culture-dependent isolation strategies revealed that *Providencia* sp. was the most dominant bacterial species detected from the guts of BSFL reared on CM and KW. *Morganella* sp. and *Brevibacterium* sp. were detected in CM, while *Staphylococcus* sp. and *Bordetella* sp. were specific to KW. However, metagenomic studies showed that *Providencia* and *Bordetella* were the dominant genera observed in BSFL gut and processed waste substrates. *Pseudomonas* and *Comamonas* were recorded in the raw waste substrates. The diversity of bacterial genera recorded from the fresh rearing substrates was significantly higher compared to the diversity observed in the gut of the BSFL and BSF frass (leftovers of the rearing substrates). These findings demonstrate that the presence and abundance of microbiota in BSFL and their associated waste vary considerably. However, the presence of clinically pathogenic strains of bacteria in the gut of BSFL fed both substrates highlight the biosafety risk of potential vertical transmission that might occur, if appropriate pre-and-postharvest measures are not enforced.

## Introduction

The expanding world population, rapid urbanization, and growing welfare have increased the demand for animal products (FAO, 2009; UN, 2014). However, the inclusion of more animal-based products constitute a major challenge for the global food production system in terms of sustainability due to the high ecological footprint associated with the production of meat and dairy (Gerber et al., 2013; Springmann et al., 2018; Kim et al., 2020). Therefore, access to affordable and innovative feed is a prerequisite to establish profitable and sustainable livestock and fisheries production systems and to ensure food security, especially in the developing world.

Recently, protein-rich edible insects have been recognized as innovative protein alternatives due to their ability in decomposing and valorizing different organic wastes (Nowak et al., 2016; Van Huis and Tomberlin, 2017; Surendra et al., 2020). Moreover, insects are rich in micronutrients, energy and fatty acids (Sheppard et al., 1994; Finke, 2013; Nowak et al., 2016). For instance, black soldier fly larvae (BSFL) *Hermetia illucens* L. (Diptera: Stratiomyidae) have been identified as a promising feed ingredient for poultry, pigs, and aquaculture (Makkar et al., 2014; Lock et al., 2018; Dörper et al., 2020; Veldkamp and Vernooij, 2021). Black soldier fly (BSF), originally traced to the Americas, is present in most tropical and temperate regions of the globe (Sheppard et al., 1994). Crude protein constitutes about 35 to 49% of the total dry weight of the BSFL while fat accounts for about 29–35% of their total dry weight and their amino acid profile is of a similar quality to that of fishmeal (Renna et al., 2017; Dabbou et al., 2018; Spranghers et al., 2018; Gasco et al., 2019). Though naturally occurring in chicken, pigs and cow manure, BSFL have been successfully reared on other organic waste streams such as catering waste, urban municipal organic waste, fish viscera, vegetable remains, coffee bean pulp, straw, and dried distillers grains with solubles (Nguyen et al., 2015; Leong et al., 2016; Van Huis and Tomberlin, 2017; Meneguz et al., 2018; Surendra et al., 2020). Moreover, BSF breeding has recently been developed at industrial scale in order to reduce large amounts of wastes produced in facilities (Miranda et al., 2020; Scala et al., 2020).

Previous studies have also reported the potential of BSFL to reduce *Escherichia coli* and *Salmonella enterica* loads in chicken and cow manure (Erickson et al., 2004; Lalander et al., 2013, 2015; Čičková et al., 2015; Nguyen et al., 2015). However, several studies revealed a considerable influence of the rearing substrate on the gut microflora of BSFL (Dillon and Dillon, 2004; Jeon et al., 2011; Engel and Moran, 2013; EFSA Scientific Committee, 2015; Boccazzi et al., 2017; Klammsteiner et al., 2018; Khamis et al., 2020). Therefore, BSFL gut may assimilate pathogens present in rearing substrates or may proliferate by inappropriate processing and storage (Bruno et al., 2019; Khamis et al., 2020). This may subsequently cause diseases in animals fed with BSFL-derived feed, highlighting the importance of selecting safe rearing substrates for the successful production of feed (Erickson et al., 2004; Čičková et al., 2015; Wang and Shelomi, 2017).

Several studies investigated the influence of rearing substrates on the dynamics of BSFL gut microflora during rearing in either a laboratory setting or in production facilities (De Smet et al., 2018; Bruno et al., 2019; Jiang et al., 2019; Wynants et al., 2019; Cifuentes et al., 2020). However, these studies were conducted in the developed world where strict regulations are imposed on production facilities (Jeon et al., 2011; Zheng et al., 2013; Boccazzi et al., 2017; Bruno et al., 2019; Wynants et al., 2019). On the other hand, little is known about the safety of BSFL-derived feeds in Africa. Therefore, the current study sought firstly to investigate the variability of the bacterial species associated with the guts of BSFL and two readily available rearing substrates in the Kenyan capital Nairobi [chicken manure (CM) and kitchen waste (KW)]. Although, both waste streams are not allowed in the European Union (Insects as Feed EU Legislation – Aquaculture, Poultry & Pig Species), their suitability and utilization for rearing BSFL is widely accepted in Africa (EFSA Scientific Committee, 2015; Nakimbugwe et al., 2020). Secondly, to investigate the bacterial species associated with BSFL frass, given their importance as organic fertilizer for crop production (Lalander et al., 2015; Oonincx et al., 2015; Beesigamukama et al., 2020).

## Materials and Methods

The study was undertaken at the International Centre for Insect Physiology and Ecology (ICIPE), Kasarani, Nairobi, Kenya (S 01_13′14.6″; E 036_53′44.5″, 1,612 m.a.s.l.).

### Stock Colony

The ICIPE Animal Rearing and Containment Unit (ARCU) maintains a population of BSF adults which acted as the stock colony for this study. The stock colony was established following methods described by Booth and Sheppard (1984) and Sripontan et al. (2017).

The adult BSF were housed in metal framed cages with screen (1.8 × 1.8 × 1.8 m with 1.5 mm mesh) with strong access to daylight spectra, while temperatures were maintained at 28 ± 5°C to encourage mating. Flies were supplied with water to prolong their life, and corrugated cardboard and some spent grain (SG) were placed within the cage to stimulate oviposition. The eggs were hatched on wheat bran and harvested for the experiment after day 5.

### Preparation of Substrate and Larvae Feeding

The two feeding substrates, CM and KW, were both sourced locally. Chicken manure was collected from a broiler poultry farm in the greater Nairobi area and fermented for 1-week to enhance moisturization. Kitchen waste was a mixture of potato peeling, carrot remains and peelings, rice and bread debris collected from a local restaurant in Nairobi. Kitchen waste materials were chopped into fine pieces and fed directly to the larvae. The feedstock was hydrated to approximately 60 ± 5% moisture by weight.

One hundred (100) 5-days neonatal BSFL obtained from ICIPE’s stock colony were placed carefully in 23 × 15 cm plastic containers containing 2 kg of the substrates. During the rearing process, the temperature was maintained at 28 ± 2°C and relative humidity at 65 ± 5%. Distilled water was sprinkled on the substrate to ensure 65–70% moisture content. The relative humidity was confirmed using a moisture sensor with two 12 cm long probes (HydroSenseTM CS620; Campbell Scientific, Inc., Logan, UT, United States). Once the substrate had been broken down by the larvae, the dried peels or coarse particles were removed. The rearing time ranged between 16 and 21 days. The fifth instar larvae were harvested and stored at −20°C to avoid any changes in the BSFL microflora until further analysis. Samples of both fresh rearing substrates (prior to the exposure to BSFL) and BSF frass (left-overs of waste substrates after exposure to BSFL) were also harvested and stored at −20°C to avoid any changes in their microflora until further analysis.

### Isolation and Morphological Characterization of Bacterial Cultures

The isolation of the larval guts was performed following aseptic techniques and under a closed sterile Purifier Logic + Class II, Type A2 Biosafety Cabinet (Labconco, Kansas City, MO, United States). The exterior of each BSF was washed once in 70% ethanol and then in 0.9% sterile phosphate-buffered saline (PBS). The entire BSFL gut was extracted using fine-tipped forceps and homogenized in a 2.0 ml microcentrifuge tubes containing 1.5 mL of sterile 0.9% PBS. Guts of a total of 50 CM fed and 50 KW fed BSFL were successfully extracted. To isolate culturable bacterial strains, aliquots of 0.1 mL from each extracted gut were spread onto agar plates containing either Nutrient Agar, MacConkey Agar or Violet Red Bile Agar (VRBA) then incubated at 37°C for 48 h. MacConkey was used to isolate coliforms and other gut pathogens particularly members of the family Enterobacteriaceae and the genus *Pseudomonas*. VRBA on the other hand was used to isolate, detect, and enumerate coli-aerogenes bacteria with emphasis being on *Enterobacter aerogenes, Escherichia coli, Salmonella* Enteritidis, *Salmonella* Typhimurium, and *Staphylococcus aureus* due to the nature of the BSF rearing substrates. Three replicates were prepared for each medium type. Selected discrete bacterial colonies were then aseptically removed by a sterile inoculation loop and sub-cultured 3 to 4 times on the same agar medium for 48 h at 37°C. The isolates were morphologically identified using traditional bacterial methods: handbooks, identification keys based on colony characteristics (size, shape, color, margin, and elevation) and microscopic morphology.

### Molecular Characterization of Bacterial Cultures

#### Extraction and Amplification of 16S rDNA

Bacterial isolates were aseptically harvested by scraping discrete bacterial colonies off the surface of cultures with a sterile inoculation loop. The genomic DNA was extracted using ISOLATE II Genomic DNA Kit (Bioline, London, United Kingdom). The resultant extracted DNA quality and quantity was assessed using NanoDrop 2000/2000c spectrophotometer (Thermo Fisher Scientific, Waltham, MA, United States). The 16S rDNA of each bacterial isolate was amplified in 30 μL volume PCR mix containing 10X PCR buffer (GenScript USA Inc, New Jersey, United States), 0.5 pmol μl^–1^ of each primer (27F 5′- AGAGTTTGATCMTGGCTCAG -3′ (Lane, 1991) and 1492R 5′- GGTTACCTTGTTACGACTT -3′ (Turner et al., 1999), 0.25 mM MgCl_2_, 0.0625 U μl^–1^ Taq DNA polymerase (GenScript USA Inc, New Jersey, United States), and 20 ng μl^–1^ of DNA template. PCR reactions were set up in a PTC 100 thermocycler (MJ Research, Gaithersburg, MD, United States). The cycling conditions involved an initial denaturation step at 95°C for 10 min, 35 cycles of a denaturation step at 94°C for 1 min, an annealing step of 52°C for 1 min and an extension step at 72°C for 1 min, followed by a final extension at 72°C for 10 min. The expected product size was 1,500 bp.

#### DNA Purification and Sequencing

The PCR products were resolved through 1% agarose gel for 1 h at 70 V (Bio-Rad model 200/2-0 power supply and wide mini-sub cell GT horizontal electrophoresis system, Bio-Rad laboratories, Inc., Hercules, CA, United States). The DNA was then visualized under UV-illumination. A KODAK Gel Logic 200 Imaging System software (Raytest GmbH, Straubenhardt, Germany) was used to photograph, analyZe, and document the gel. Following the manufacturer’s instructions, the QuickClean 5M Gel Extraction Kit II from GenScript (GenScript Corporation, Piscataway, NJ, United States) was used to purify the PCR products which were then sent to Macrogen Europe BV for bi-directional sequencing.

#### 16S rDNA Amplification, MinION Library Preparation and Sequencing

Genomic DNA extraction from BSFL, microbial cultures of the raw substrates, bacterial plates and frass was done using the Isolate II DNA extraction kit (Bioline, London, United Kingdom) as per the manufacturer’s protocol. The purity and concentration of the resultant DNA was determined using a Nanodrop 2000/2000c spectrophotometer (Thermo Fischer Scientific, Wilmington, NC, United States). Library preparation was performed using the Ligation Sequencing Kit (SQK-LSK108) and Native Barcoding Kit (EXP-NBD103) for genomic DNA, according to the standard 1D Native barcoding protocol provided by the manufacturer (Oxford Nanopore Technologies, Oxford, United Kingdom). The constructed library was loaded into the Flow Cell R9.4 (FLO-MIN106) of a MinION device (Oxford Nanopore Technologies, Oxford, United Kingdom), which was run with the SQK-LSK108_plus_Basecaller script of the MinKNOW1.7.14 software, for 4 h.

#### Morphological Data Analysis

Stata (version 15.1) was used for analyses. Bacterial isolates occurrence was expressed as a percentage of the total number of dissected BSFL. A two-sample test of proportions (Z-test) for non-parametric data was used to compare the occurrence of bacterial isolates obtained from larvae guts of BSF previously reared on CM and KW substrates.

#### Molecular Data Analysis

The sequences were assembled and edited using Chromas version 2.13 (Technelysium Pty Ltd, Queensland, Australia). Consensus sequences from both the forward and reverse strands were generated and were then queried through BLASTN in the GenBank database hosted by the National Center of Biotechnology Information (NCBI)^1^ for identification purposes and to check for similarity with organisms already identified. Any isolate exhibiting 97--100% sequence similarity to NCBI strains were considered as the correct species for that isolate. Moreover, the consensus sequences were aligned using ClustalX version 1.81. Sequences’ similarities and identities was done using SIAS (Sequence Identity And Similarity)^2^. Further phylogenetic analyses that were conducted using MEGA X (Kumar et al., 2018). Out of 24 nucleotide models, Kimura-2 parameter was considered the best fit model (K2 + G) for our data set since the model posted the lowest Bayesian Information Criterion. The evolutionary history was inferred by using the Maximum Likelihood method and Kimura 2-parameter model (Kimura, 1980). The tree with the highest log likelihood (−18732.10) is shown. The percentage of trees in which the associated taxa clustered together is shown next to the branches. Initial tree(s) for the heuristic search were obtained automatically by applying Neighbor-Join and BioNJ algorithms to a matrix of pairwise distances estimated using the Maximum Composite Likelihood (MCL) approach, and then selecting the topology with superior log likelihood value. The tree is drawn to scale, with branch lengths measured in the number of substitutions per site. This analysis involved 53 nucleotide sequences. Codon positions included were 1st + 2nd + 3rd + Non-coding. There were a total of 1977 positions in the final dataset.

#### Metagenomic Data Analysis

Low quality sequence reads, primers and adapters were trimmed from the sequences using the trim.seqs script in Mothur (1.30.2) (Schloss et al., 2009). Reads were formatted and chimeras removed using chimera.slayer function in Mothur and the paired- end reads merged using FLASH (v1.2.7) (Magoc and Salzberg, 2011). High- quality sequences were aligned using INFERNAL aligner at the Ribosomal Database Project (RDP) website with a threshold bootstrap of 80% (Cole et al., 2009). Resulting data were analyzed using QIIME 2 where the sequences were used to pick Operational Taxonomic Units (OTUs) at 97% similarity threshold (Caporaso et al., 2010) and taxonomy assigned using RDP classifier using Greengenes database as the reference database (McDonald et al., 2012). Alpha diversity runs were done to calculate species richness and species diversity using the Shannon indexing (Spellerberg and Fedor, 2003) with rarefaction curves generated to describe OTU abundance and diversity in QIIME. Stacked bar plot was used to do quantitative comparison on abundance at genus level with a minimum abundance of 0.1% cut off being set to select the most abundant taxa in each sample. Any taxa with read counts below the 0.1% threshold was collapsed and lumped into the “others” category. In a community, if the bacterial species numbers (both culturable and non-culturable) are large and diversified, viewing at genus level (higher levels) provides better picture as compared to lower levels (i.e., species level). For microbial comparison between the different groups of samples, Beta diversity was calculated using UNIFRAC metrics (Lozupone et al., 2011). Principle Coordinate Analysis (PCoA) was used to visualize the differences between the microbial communities and heat maps and/or rarefaction curves generated.

For the microbiome data, differences in intrinsic parameters such as OTU richness and diversity parameters were analyzed using ANOVA or ANOSIM (analysis of similarity) in R Studio (version 3.2.5) (R Development Core Team, 2016). For all tests a significant level of 0.05 was considered. Non-metric multidimensional scaling (NMDS) was also done using R package vegan (v.2.43) to create dissimilarity matrixes using the Bray-Curtis dissimilarity method. All raw data were deposited in the NCBI database as BioProject:PRJNA728669 and BioSample SAMN19093411.

## Results

### Morphological Identification of Bacterial Isolates

**FIGURE 1:**
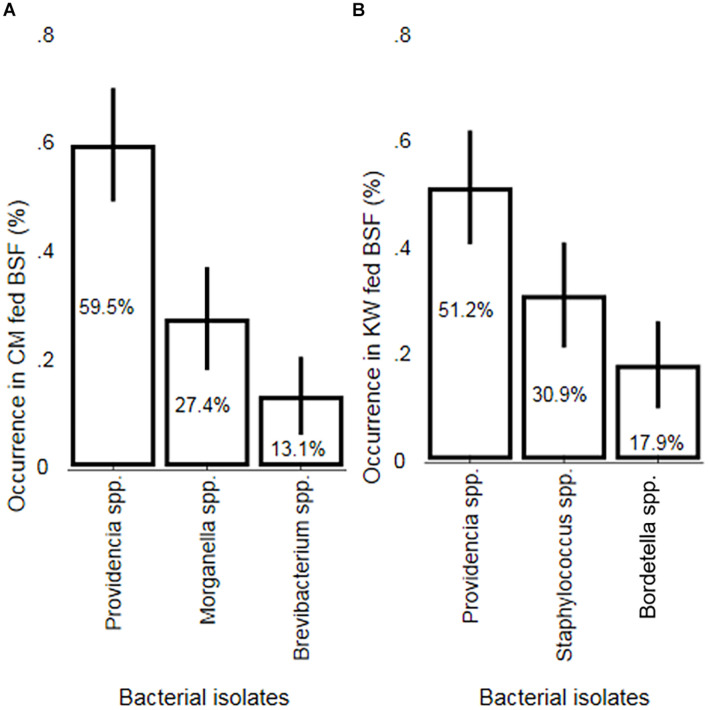
A two-sample test of proportions (Z-test) for non-parametric data depicting the occurrence of bacterial isolates obtained from larvae guts of BSF previously reared on CM and KW substrates. Mean bacterial isolates occurrence (in%) in the gut of **(A)** chicken manure (CM) and **(B)** kitchen waste (KW) fed black soldier fly larvae (BSFL). Error bars represent 95% confidence intervals.

A total of five bacteria isolates belonging to the genera *Providencia*, *Morganella*, *Brevibacterium*, *Staphylococcus* and *Bordetella* were obtained from the 50 dissected BSFL reared on CM and KW. *Providencia* was the most abundant genus in the guts of BSFL reared on CM (59.5%) and KW (51.2%) [95% confidence interval (CI) 49.0–70.0% and 95% CI 40.5–61.9%, respectively]. Both *Morganella* and *Brevibacterium* occurred only in BSFL reared on CM at 27.4% [95% CI 17.9–36.9%] and 13.1% [95% CI 5.9–20.3%], respectively. Moreover, *Staphylococcus* and *Bordetella* both occurred only in BSFL reared on KW at 30.9% [95% CI 21.1–40.8%] and 17.9% [95% CI 9.7–26.1%], respectively (Figure 1). We found a significant difference in the occurrence of *Providencia* obtained from BSFL reared on CM in comparison to both *Morganella* (*p* = 0.001) and *Brevibacterium* (*p* < 0.001). Moreover, there was a significant difference between the occurrence of *Morganella* and *Brevibacterium* (*p* = 0.035). In addition, the occurrence of *Providencia* obtained from BSFL reared on KW fed in comparison to both *Staphylococcus* (*p* = 0.036) and *Bordetella* (*p* < 0.001) differed significantly. On the other hand, no significant difference was found between the occurrence of *Staphylococcus* and *Bordetella* (*p* = 0.080).

GenBank accession numbers provided for the nucleotide sequences of the bacterial isolates are as follows: *Providencia* sp. MSB6 = MK276967, *Providencia* sp. MSB9 = MK276968, *Providencia* sp. MSB12 = MK276969, *Providencia* sp. MSB22 = MK276974, *Morganella* sp. MSB27 = MK276976, *Brevibacterium* sp. MSB14 = MK276970, *Staphylococcus* sp. MSB18 = MK276972, *Bordetella* sp. MSB17 = MK276971, *Bordetella* sp. MSB21 = MK276973, and *Bordetella* sp. MSB24 = MK276975.

### Molecular Characterization of Bacterial Isolates

The isolated bacterial species depicting isolate species identities with 97–98% similarity and 0.0 E values after sequencing have been molecularly identified (Table 1). Like the morphological identification, the molecular characterization also yielded five isolate species identified as *Providencia rettgeri* (isolates MSB6, MSB9, MSB12, and MSB22), *Morganella morganii* (isolate MSB27), *Brevibacterium luteolum* (isolate MSB14), *Staphylococcus* sp. (isolate MSB18) and *Bordetella* sp. (isolates MSB17, MSB21, and MSB24) (Supplementary Table 2). Thus, the molecular identification of the bacterial isolates corroborates that of the morphological study (Figure 1 and Table 1).

**TABLE 1:** Summary of the identified bacterial species using 27 F and 1492 R primers.

**Bacterial isolates codes**	**Bacterial isolates sources**	**ID from GeneBank**	**ID %**	**Species GenBank accession number**
MSB6	BSF reared on CM	*Providencia rettgeri* strain ALK417 16S	97	KC456547.1
MSB9	BSF reared on CM	*Providencia rettgeri* strain ALK417 16S	97	KC456547.1
MSB12	BSF reared on CM	*Providencia rettgeri* strain ALK417 16S	98	KC456547.1
MSB22	BSF reared on KW	*Providencia rettgeri* strain ALK417 16S	98	KC456547.1
MSB27	BSF reared on CM	*Morganella morganii* subsp. *morganii* strain ALK057 16S	97	KC456563.1
MSB14	BSF reared on CM	*Brevibacterium luteolum* partial 16S rRNA	97	LT222061.1
MSB18	BSF reared on KW	*Staphylococcus* sp. strain 82584 16S	98	KX525724.1
MSB17	BSF reared on KW	*Bordetella* sp. BAB-4401 16S	98	KP751929.1
MSB21	BSF reared on KW	*Bordetella* sp. BAB-4401 16S	97	KP751929.1
MSB24	BSF reared on KW	*Bordetella* sp. BAB-4401 16S	98	KP751929.1

**FIGURE 2:**
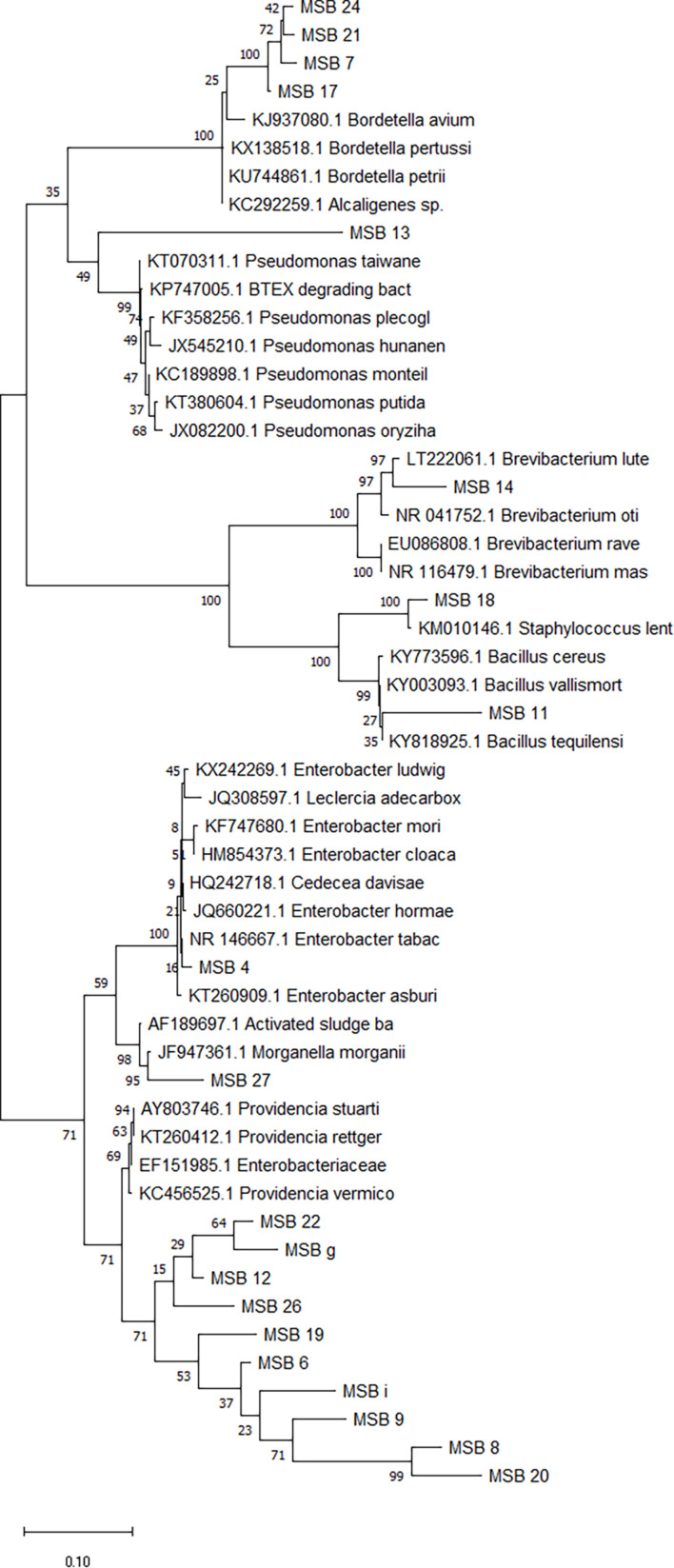
Phylogenetic tree by Maximum Likelihood Method showing the evolutionary relationships of bacterial isolates from chicken manure (CM) and kitchen waste (KW) fed black soldier fly larvae (BSFL) and isolates from GenBank.

The evolutionary history of the bacterial isolates was inferred using the Neighbor-Joining method (Saitou and Nei, 1987). The optimal tree with the sum of branch length = 0.52129017 is presented in Figure 2. In this figure the percentage of replicate trees in which the associated taxa clustered together in the bootstrap test (500 replicates) is shown next to the branches (Felsenstein, 1985). The tree is drawn to scale, with branch lengths in the same units as those of the evolutionary distances used to infer the phylogenetic tree (Figure 2). The analysis involved 15 nucleotide sequences. Codon positions included were 1st + 2nd + 3rd + Non-coding. All positions containing gaps and missing data were eliminated. There was a total of 966 positions in the final dataset. Five groups resulted from this analysis (Figure 2). The first group consisted of the *Providencia* sp. isolates where each of the samples MSB12, MSB22, MSB6, and MSB9 isolates branched separately. Furthermore, all the *Providencia* isolates linked to *Providencia rettgeri* of accession number KC456547.1 during blasting (Table 1). The second group consisted of *Morganella* sp. isolate MSB27 which linked to *Morganella morganii* of accession number KC456563.1 during blasting. The third group consisted of *Brevibacterium* sp. isolate MSB14 which linked to *Brevibacterium luteolum* of LT222061.1 during blasting. The last two clusters of the phylogenetic tree consisted of *Staphylococcus* sp. isolate MSB18 and *Bordetella* sp. isolates MSB17, MSB21, and MSB24, respectively (Figure 2). The *Staphylococcus* sp. isolate linked to *Staphylococcus* sp. accession number KX525724.1 while all the *Bordetella* isolates linked to *Bordetella* sp. of accession number KP751929.1 during blasting (Table 1).

### Library Size and Cumulative Abundance

**FIGURE 3:**
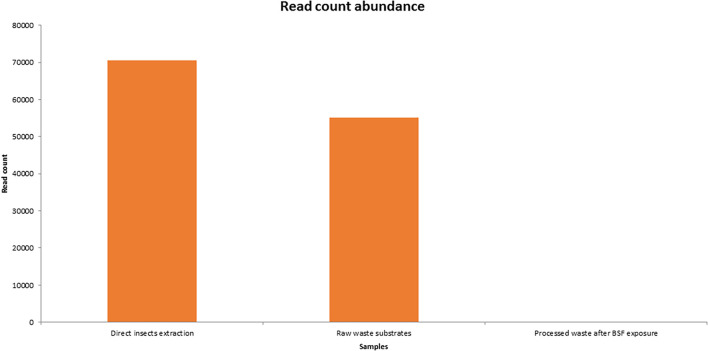
Overview of 16S metagenomic library size generated by MinION sequencing of BSF and its substrates. There were three pooled sample categories i.e., direct insects extraction batch, raw waste substrates, and the processed waste substrates after BSF exposures.

**FIGURE 4:**
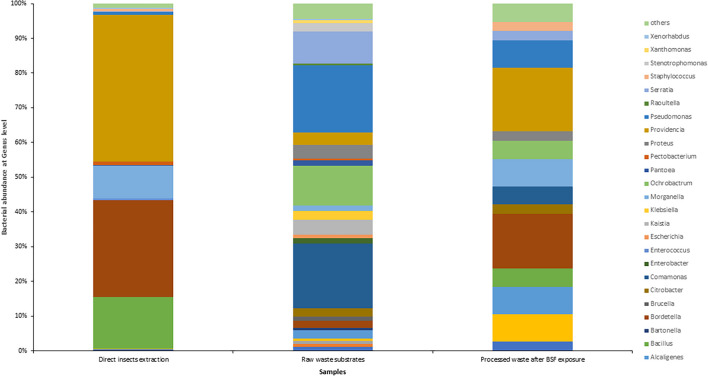
Taxonomic composition and percentage of reads of bacteria at the genus level, in the black soldier fly larvae (BSFL) and its substrates, using Stacked Bar plot. For clarity, taxa with cumulative read counts below the cut-off value of 0.1% were summed into “Others” category.

The insect extraction had the largest 16S metagenomic library size of 70,518 reads, followed by the fresh waste substrates (55,184 reads) then finally the BSF frass exposures at 38 reads (Figure 3). The taxonomic composition at genus levels of the samples and the cumulative abundance of the bacterial genomes is detailed in Figure 4. *Providencia* (42.28%) and *Bordetella* (27.75%) were the most abundant genera in the direct insects’ extraction batch (non- cultured samples). In the raw waste substrates, *Pseudomonas* (19.3%) and *Comamonas* (18.7%) were most abundant genera while *Providencia* (18.42%) and *Bordetella* (15.8%) were most abundant genera in the BSFL frass (Figure 4).

### Alpha Diversity and Beta Diversity Analyses

The fresh waste substrates had the highest species richness (30), followed by the direct BSFL extracts (26) and the BSFL frass (15) (Figure 4 and Supplementary Table 1). In terms of homogeneity, BSFL frass had the highest evenness index (Figure 4 and Supplementary Table 5) while BSFL extract was the most heterogeneous.

**FIGURE 5:**
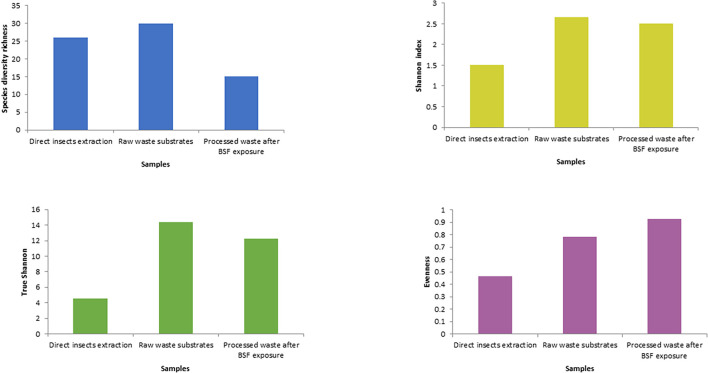
Alpha-diversity measures using evenness, Shannon diversity index, and species richness at the genus level in the black soldier fly larvae (BSFL) and its substrates. The samples were composed grouped into three pooled categories (insects’ extraction, raw waste substrates, and the BSF frass) and are represented on the *X*-axis, and their estimated diversity is represented on the *Y*-axis.

**FIGURE 6:**
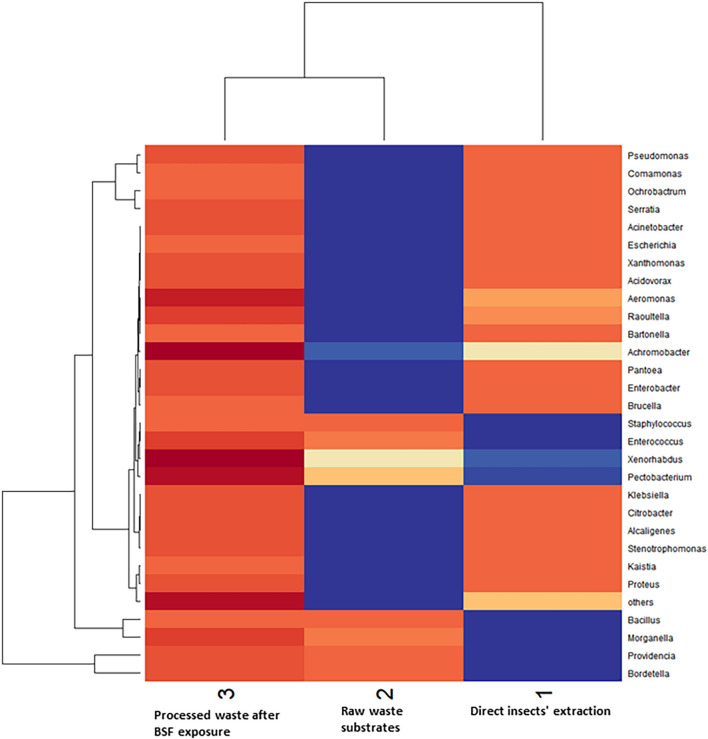
A heatmap depicts the differential abundance of microbial taxa that varied among sample groups at FDR < 0.05. Rows (microbial taxa at genus level) and columns (samples) were ordered by hierarchical clustering. The top dendrogram shows which environmental parameters (samples) have the most similar responses. The side dendrogram shows which genera are behaving most similar.

The Shannon diversity index showed that raw waste substrates had the highest diversity of bacterial genera (2.67), followed by BSFL frass (2.5), and direct BSFL extract (1.5) (Figure 5). The effective diversity (True-Shannon) followed the same trend and showed that the raw waste substrates had the highest effective number of species (14.38), followed by BSFL frass (12.22), and BSFL extract (4.54) (Figure 5 and Supplementary Table 3). A heatmap (Figure 6) depicting the differential abundance of microbial taxa that varied among sample groups at FDR < 0.05 was generated.

**FIGURE 7:**
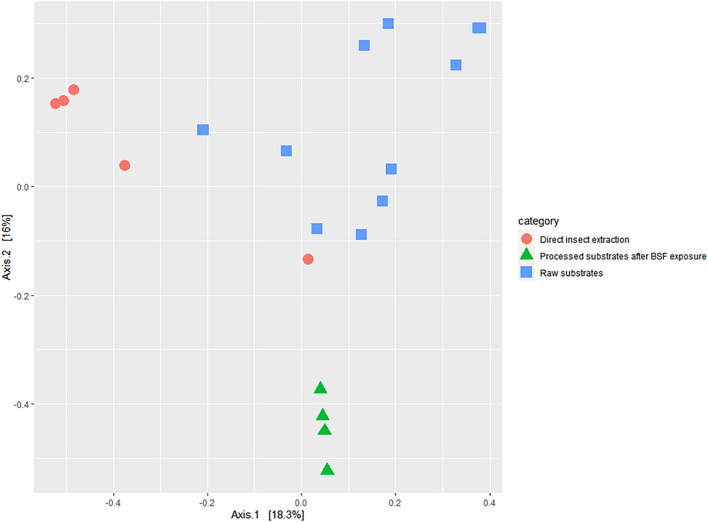
Two-dimensional principal coordinate analyses plot of the beta diversity of bacterial genera in the black soldier fly larvae (BSFL) and its substrates, estimated using the Bray Curtis dissimilarity index showing.

According to the Bray Curtis dissimilarity index, the interpopulation diversity index between BSFL extracts, and BSFL frass at its lowest was 89.62% (Figure 7 and Supplementary Table 6).

## Discussion

Chicken manure and kitchen waste are two common organic waste streams in Nairobi and arguably also in other megacities in the world (Shumo et al., 2019b). We reared BSFL on these waste streams and used both a culture-dependent and independent sequence-based approach to survey the bacterial species in the larval gut. The effect of these waste streams on the bacterial communities of BSFL and the BSFL frass were also evaluated.

Bacteria of the genus *Providencia* are Gram-negative opportunistic pathogens have been isolated from a wide variety of environments and organisms, ranging from humans to insects, sea turtles and shark mouths (Ami et al., 2010; Galac and Lazzaro, 2011; Hamden et al., 2013; Augustinos et al., 2015). In addition, *Providencia* spp. are associated with a wide range of human infections, and they show harmful and pathogenic effects on their hosts. This may have an economic impact on the food safety industry. However, *Providencia* are known to be vertically transmitted in BSF and can stimulate oviposition (Galac and Lazzaro, 2011; De Smet et al., 2018). *Morganella* sp. belonging to the phylum Proteobacteria and *Brevibacterium* spp. belonging to the phylum Actinobacteria were both detected only in CM fed BSFL. In contrast, *Staphylococcus* sp. (phylum Firmicutes) and *Bordetella* sp. (phylum Proteobacteria) were only detected in KW fed BSFL. This reconfirms the results reported by Osimani et al. (2021) which detected an influence of the rearing substrate on of the relative abundance of *Morganella* sp. in BSFL. *Staphylococcus* sp. are Gram-positive bacteria and are commonly found as symbionts in the guts of different insect species like the common fruit fly *Drospholia melanogaster* Meigen (Diptera: Drosophilidae), the southern house mosquito *Culex quinquesfasciatus* Say (Diptera: Culicidae), *Analeptes trifasciata* Fabr. (Coleoptera: Cerambycidae) and the drosophila parasitoid wasp *Asobara tabida* (Nees) (Hymenoptera: Braconidae) (Zouache et al., 2009; Oyedokun and Adeniyi, 2016). *Staphylococci* are well known for developing antibiotic resistance as well as causing food-borne diseases and nosocomial infections (Kadariya et al., 2014).

Our metagenomics study confirms the presence of several species including *Morganella*, *Enterococcus*, *Pseudomonas* and *Providencia* that were previously reported (Klammsteiner et al., 2018; Bruno et al., 2019; Khamis et al., 2020; Tanga et al., 2021). The presence of universal bacterial species across studies, substrate and locations may indicate the existence of conserved members of the BSF larval gut microbiota. However, no studies have investigated the interactive roles of these conserved members with their associated BSFL host. Obtaining such information would be important in determining whether the BSFL have a core gut microbiota, that might be important in enhancing their performance or vice versa. However, what remains puzzling to us is the absence of *Dysgonomonas*. Previous studies reported *Dysgonomonas* as one of the top three most abundant members of the BSFL gut microbiota (Klammsteiner et al., 2018; Bruno et al., 2019; Khamis et al., 2020; Tanga et al., 2021). *Dysgonomonas* sp. is widely known for its essential role in the gut of termites during the degradation of recalcitrant lignocellulose (Yang et al., 2014; Sun et al., 2015). Bruno et al. (2019) reported that *Dysgonomonas* plays a significant role in the digestion of complex polysaccharides. This was reconfirmed as Jiang et al. (2019) reported that *Dysgonomonas* sp. obtained from the gut of BSF is directly associated with genes for sulphate, carbohydrate, and nitrogen metabolism. Moreover, a metagenomic analysis of the BSFL gut traced the origin of a new α-galactosidase gene that facilitates the breaking up of α-galactoses abundant in non-digestible plant carbohydrates to a specific *Dysgonomonas* strain (Lee et al., 2018). Additionally, *Dysgonomonas* sp. may contribute to the biodegradation of pharmaceutical products like ciprofloxacin when appearing in a consortium with other bacteria-like microorganisms, for instance, *Actinomyces* sp., underlining the potential for further biotechnological applications (Martins et al., 2018).

In our study, *Bacillus* sp. was observed in BSFL extracts and members of this genus are known for their wide range of physiologic characteristics and their ability to produce enzymes, antibiotics, and metabolites. That explains why they have been widely used in many medical, pharmaceutical, agricultural, and industrial processes (Logan, 2012; EFSA Panel on Biological Hazards [BIOHAZ], 2016; Celandroni et al., 2019). In addition, they produce nutraceuticals such as vitamins such as riboflavin, cobalamin, and inositol as well as carotenoids. For that reason, they are used in the production of several health supplements for human consumption (Mohammed et al., 2014; Tanaka et al., 2014; Takano, 2016).

Although the BSFL used in this study and the ones used in previous study by Tanga et al. (2021) were obtained from the same stock colony, bacterial species reported in both studies showed a high degree of variability. This suggests that despite the possibility of the presence of a BSF core gut microbiota, other factors may have contributed to the microflora present. These factors may include the organic waste streams used (diet), biotic and abiotic factors. For instance, Tanga et al. (2021) reported the presence of gastrointestinal pathogens *Campylobacter*, which might be attributed to external contamination of the rearing substrates before the BSFL were introduced. Likewise, Jeon et al. (2011) reported a higher bacterial diversity of food waste fed larvae in comparison to ones reared on cooked rice or calf forage; the bacterial variability was attributed to the influence of the rearing substrates. Moreover, environmental conditions probably play an important role in shaping the microbial communities in the guts of BSFL. For instance, oxygen variability can influence the gut shape which in return influences the microbial communities inhabiting the gut (Hoback and Stanley, 2001; Ke et al., 2010). Moreover, the pH content of the rearing substrates possibly facilitates the presence of certain bacterial species and inhibits the growth of others. Erickson et al. (2004) observed a reduction in pathogenic bacterial populations in alkaline BSFL rearing substrates such as CM in comparison to acidic ones like cow or hog manure. Moreover, temperature may have an influence on the bacterial composition of the rearing substrates and consequently on the BSFL gut microflora. Studies on the potential of BSFL in the reduction of *Escherichia coli* and *Salmonella* spp. showed that both the nature of the rearing substrate as well as the temperature may influence the effectiveness of such a reduction. For instance, Erickson et al. (2004) reported that the effectiveness of BSFL in the reduction of *Escherichia coli* and *Salmonella* spp. increased with increasing temperatures as they observed higher reduction rates at temperatures of 27°C and 32°C in comparison to 23°C. Likewise, Liu et al. (2008) demonstrated an increase in the effectiveness of BSFL in the reduction of *Escherichia coli* and *Salmonella* spp. in cow manure accompanied with a 100% mortality of BSFL at a temperature of 35°C. However, they observed a similar increase in the reduction of bacterial counts in cow manure controls not containing BSFL at the same temperature. Yet, Shumo et al. (2019a) found that BSFL were capable of surviving at higher temperatures (up to 35°C), which is an indication of phenotypic plasticity. This has also been reported by Moczek (2010) and Kelly et al. (2012). Therefore, further studies are needed to verify whether phenotypic plasticity influences the dynamics of microbial communities associated with the guts of BSFL.

The presence of certain bacterial species may also be linked to vertical transmission as reported by Su et al. (2010), who demonstrated the presence of both *Providencia* spp. and *Morganella morganii* in the gut of newly emerged adult house flies. This indicates that certain microorganisms are vertically transmitted and can be carried over in the gut from the larval to adult stage. Moreover, Zheng et al. (2013) recorded *Providencia* spp. in both eggs and adult BSF. Insects have developed different mechanisms for vertical transmission to ensure that offspring acquires the necessary microbial symbionts (Engel and Moran, 2013). Symbiotic microorganisms are important for host survival and reproduction as they perform essential metabolic roles such as nutrient digestion (Engel and Moran, 2013). For example, *Providencia* spp. and *Morganella morganii* can express urease which in turn leads to the production of high levels of biogenic amines (Hu et al., 1990; Zogul and Zogul, 2004). Biogenic amines neutralizes acidic digestive fluids in the host’s gut and therefore prevents the hydrolysis of certain bacterial species (Kawahara et al., 1999). Moreover, the presence of bacteria may be influenced by shifts in the insect life cycle. In our study, the BSFL guts were examined only at the fifth instar larval stage. Holometabolous insects such as BSF experience metabolically dynamic and complex processes during their transition from larvae to adults (Kohl et al., 2013), and gut microbial communities experience significant changes during metamorphosis and in the adult stage (Moll et al., 2001). Likely the gut undergoes a sterilization process during metamorphosis and acquires new microbiota in the adult stage (Kohl et al., 2013). Yet, Chen et al. (2016) showed that *Enterococcus* spp. such as *Enterococcus mundtii* survived metamorphosis in the gut of the cotton leafworm *Spodoptera littoralis* (Boisduval) (Lepidoptera: Noctuidae) and were carried over to the adult stage. Moreover, Zheng et al. (2013) reported shifts in the gut bacterial composition of BSF reared on identical rearing substrates during different development stages. Finally, Su et al. (2010) reported a carry-over of several bacterial taxa through larval stages to newly emerged adult house flies. Such observations suggest that though the composition of microbial communities associated with the BSF gut undergoes changes during metamorphosis, certain bacterial species are carried over regardless of life stages development. The wide-spread presence of these bacterial genera in different BSFL from different locations suggest the possible existence of core microbiota in BSF. Yet, the abundance of these species seems highly variable depending on the abiotic and biotic factors in the rearing system, and possibly even the insect strain (Wynants et al., 2019).

In our study, the diversity of bacterial species in BSFL extract was relatively low in comparison to fresh rearing substrates and BSF frass. Additionally, *Staphylococcus* sp. was present in limited amounts in BSFL extracts in comparison to fresh rearing substrates and BSF frass. *Staphylococcus* spp. is a genus of gram-positive bacteria that colonizes a variety of animal species (Enright et al., 2002; Sung et al., 2008; Sakwinska et al., 2011). Almost all staphylococcal species have been identified as causes of opportunistic infections (Otto, 2004). However, certain *Staphylococcus* species are recurring and are considered threatening pathogens (Weese, 2010). For instance, *Staphylococcus aureus* is a dangerous pathogen that can cause severe and life-threatening human diseases including severe sepsis, pneumonia, toxic shock syndrome and endocarditis (Lowy, 1998). Moreover, *Staphylococcus aureus* can cause infections in animals including mastitis in dairy-producing animals and bumblefoot in chickens (McNamee and Smyth, 2000; Dufour et al., 2012; Zecconi and Scali, 2013). However, the significance of *Staphylococcus aureus* in terms of public health is caused by its ability to develop resistance to antimicrobials (Weese, 2010). The low diversity and limited abundance of bacterial species including *Staphylococcus* sp. that we observed in our study may be attributed to the expression of antimicrobial peptides (AMPs) in BSFL. AMPs are pathogen-targeting peptides that are produced in fat and blood cells and secreted to hemolymph through the activation of humoral immunity in response to pathogenic infections (Boman, 1995; Bulet et al., 1999; Brogden, 2005). AMPS which are small cationic molecules composed of 10 to 100 amino acids exhibit activities against bacteria, fungi, viruses and parasites (Lavine and Strand, 2002). Moreover, certain AMPS exhibit a cytotoxic behavior toward cancer cells (Hoskin and Ramamoorthy, 2008). Previous studies reported that insects including Coleoptera, Diptera, Hymenoptera and Lepidoptera can abundantly produce AMPs (Casteels et al., 1990; Bulet et al., 1999; Hoffmann and Reichhart, 2002; Goo et al., 2008a,b; Vetterli et al., 2018). For instance, a study by Choi et al. (2012) reported that a methanol extract of BSFL demonstrated antibacterial effects against gram-negative bacteria. Moreover, rearing BSFL on contaminated organic waste streams in our study may have enhanced the production of AMPs. Previous studies confirmed that the production of AMPs in insects is enhanced by bacterial infections (Lemaitre et al., 1997; Bulet et al., 1999; Hoffmann and Reichhart, 2002; Wang et al., 2016; Wu et al., 2018). Specifically, several studies reported a significant increase in the expression of AMPs in BSFL infected with bacteria (Vieira et al., 2014; Park et al., 2016; Lee et al., 2020). These results suggest that BSFL may be used in the commercial production of natural antibiotics that can replace synthetic ones in the future (Bahar and Ren, 2013). The replacement of synthetic antibiotics with natural ones can put an end to antibiotics resistance and antibiotics related environment pollution (Lee et al., 2020). Therefore, future research should focus on the possibility of developing cost effective and biologically stable BSFL- derived AMPs extraction and production methods.

## Conclusion and Outlook

Even though this study has demonstrated the influence of rearing substrates on the gut microbial community of BSFL and indicated the potential of BSFL to up-take, among others, pathogens from contaminated rearing substrates, further assays need to be undertaken over a period to ascertain the trends observed. Moreover, the dominant presence of *Providencia* spp. in the guts of BSFL reared on both substrates highlights the existence of a core microbiota in their guts, irrespective of the rearing substrates used and needs to be explored further over time. The presence of some clinically pathogenic bacteria in the gut of BSFL is an indication that the selection of safe organic waste streams for industrial production of BSFL production for the wide market is crucial. This scenario is not common to the African continent alone, therefore global insect-based feed policies should consider such findings. To conclude, policies and regulations that govern the production and use of emerging insect meal as an alternative to conventional meal sources as well as appropriate safety hygiene and quality control standards needs to be developed.

## Data Availability Statement

The data for morphological identification of bacterial isolates presented in the study are deposited in the GenBank of the National Center for Biotechnology Information (NCBI) repository (https://www.ncbi.nlm.nih.gov/nuccore/). Accession numbers provided for the nucleotide sequences of the bacterial isolates are as follows: *Providencia* sp. MSB6 = MK276967, *Providencia* sp. MSB9 = MK276968, *Providencia* sp. MSB12 = MK276969, *Providencia* sp. MSB22 = MK276974, *Morganella* sp. MSB27 = MK276976, *Brevibacterium* sp. MSB14 = MK276970, *Staphylococcus* sp. MSB18 = MK276972, *Bordetella* sp. MSB17 = MK276971, *Bordetella* sp. MSB21 = MK276973, and *Bordetella* sp. MSB24 = MK276975. The data for molecular data analysis presented in the study are deposited in the NCBI database repository under accession numbers PRJNA728669 (https://www.ncbi.nlm.nih.gov/bioproject/PRJNA728669) and SAMN19093411 (https://www.ncbi.nlm.nih.gov/biosample/?term=SAMN19093411).

## Author Contributions

MS, FK, FO, CT, KF, SS, SE, and CB conceived and designed the study, and discussed the results. MS performed the experiments, analyzed the data, and did the original draft preparation. FK, FO, CT, KF, SS, SE, OS, AH, and CB reviewed and edited the article. All authors contributed to the article and approved the submitted version.

## Conflict of Interest

MS was employed by the company Hermetia Baruth GmbH. The remaining authors declare that the research was conducted in the absence of any commercial or financial relationships that could be construed as a potential conflict of interest.

## Publisher’s Note

All claims expressed in this article are solely those of the authors and do not necessarily represent those of their affiliated organizations, or those of the publisher, the editors and the reviewers. Any product that may be evaluated in this article, or claim that may be made by its manufacturer, is not guaranteed or endorsed by the publisher.

## Footnotes

1
http://www.ncbi.nlm.nih.gov

2
http://imed.med.ucm.es/Tools/sias.html
