# Supplementary material for: A Molecular Survey of Bacterial Species in the Guts of Black Soldier Fly Larvae (Hermetia illucens) Reared on Two Urban Organic Waste Streams in Kenya
Source: Front Microbiol. 2021 Sep 22;12:687103. doi: 10.3389/fmicb.2021.687103 (PMC8493336; doi:10.3389/fmicb.2021.687103)
Supplement: Supplementary file 1 [file Data_Sheet_1.zip › Table 4.DOCX]

**Table. Maximum Likelihood fits of 24 different nucleotide substitution models**

| **Model** | **Parameters** | **BIC** | **AICc** | ***lnL*** | **(+*I*)** | **(+*G*)** | ***R*** | ***f*(A)** | ***f*(T)** | ***f*(C)** | ***f*(G)** | ***r*(AT)** | ***r*(AC)** | ***r*(AG)** | ***r*(TA)** | ***r*(TC)** | ***r*(TG)** | ***r*(CA)** | ***r*(CT)** | ***r*(CG)** | ***r*(GA)** | ***r*(GT)** | ***r*(GC)** |
| --- | --- | --- | --- | --- | --- | --- | --- | --- | --- | --- | --- | --- | --- | --- | --- | --- | --- | --- | --- | --- | --- | --- | --- |
| K2+G | 105 | 35675.662 | 34705.877 | -17247.792 | n/a | 0.30 | 0.90 | 0.250 | 0.250 | 0.250 | 0.250 | 0.066 | 0.066 | 0.119 | 0.066 | 0.119 | 0.066 | 0.066 | 0.119 | 0.066 | 0.119 | 0.066 | 0.066 |
| K2+G+I | 106 | 35686.912 | 34707.893 | -17247.797 | 0.00 | 0.30 | 0.90 | 0.250 | 0.250 | 0.250 | 0.250 | 0.066 | 0.066 | 0.119 | 0.066 | 0.119 | 0.066 | 0.066 | 0.119 | 0.066 | 0.119 | 0.066 | 0.066 |
| TN93+G | 109 | 35697.669 | 34690.951 | -17236.317 | n/a | 0.30 | 0.91 | 0.250 | 0.208 | 0.229 | 0.313 | 0.054 | 0.060 | 0.113 | 0.065 | 0.144 | 0.082 | 0.065 | 0.131 | 0.082 | 0.090 | 0.054 | 0.060 |
| T92+G | 106 | 35705.191 | 34726.173 | -17256.937 | n/a | 0.30 | 0.90 | 0.229 | 0.229 | 0.271 | 0.271 | 0.060 | 0.071 | 0.129 | 0.060 | 0.129 | 0.071 | 0.060 | 0.109 | 0.071 | 0.109 | 0.060 | 0.071 |
| TN93+G+I | 110 | 35708.909 | 34692.958 | -17236.318 | 0.00 | 0.30 | 0.91 | 0.250 | 0.208 | 0.229 | 0.313 | 0.054 | 0.060 | 0.113 | 0.065 | 0.144 | 0.082 | 0.065 | 0.131 | 0.082 | 0.090 | 0.054 | 0.060 |
| GTR+G | 112 | 35710.721 | 34676.304 | -17225.985 | n/a | 0.30 | 0.90 | 0.250 | 0.208 | 0.229 | 0.313 | 0.069 | 0.053 | 0.113 | 0.083 | 0.143 | 0.086 | 0.057 | 0.130 | 0.069 | 0.090 | 0.057 | 0.051 |
| T92+G+I | 107 | 35716.431 | 34728.179 | -17256.937 | 0.00 | 0.30 | 0.90 | 0.229 | 0.229 | 0.271 | 0.271 | 0.060 | 0.071 | 0.129 | 0.060 | 0.129 | 0.071 | 0.060 | 0.109 | 0.071 | 0.109 | 0.060 | 0.071 |
| GTR+G+I | 113 | 35721.961 | 34678.311 | -17225.986 | 0.00 | 0.30 | 0.90 | 0.250 | 0.208 | 0.229 | 0.313 | 0.069 | 0.053 | 0.113 | 0.083 | 0.143 | 0.086 | 0.057 | 0.130 | 0.069 | 0.090 | 0.057 | 0.051 |
| HKY+G | 108 | 35739.254 | 34741.769 | -17262.730 | n/a | 0.30 | 0.92 | 0.250 | 0.208 | 0.229 | 0.313 | 0.055 | 0.060 | 0.148 | 0.066 | 0.108 | 0.083 | 0.066 | 0.098 | 0.083 | 0.118 | 0.055 | 0.060 |
| HKY+G+I | 109 | 35750.497 | 34743.779 | -17262.732 | 0.00 | 0.30 | 0.92 | 0.250 | 0.208 | 0.229 | 0.313 | 0.055 | 0.060 | 0.148 | 0.066 | 0.108 | 0.083 | 0.066 | 0.098 | 0.083 | 0.118 | 0.055 | 0.060 |
| JC+G | 104 | 35826.820 | 34866.268 | -17328.990 | n/a | 0.30 | 0.50 | 0.250 | 0.250 | 0.250 | 0.250 | 0.083 | 0.083 | 0.083 | 0.083 | 0.083 | 0.083 | 0.083 | 0.083 | 0.083 | 0.083 | 0.083 | 0.083 |
| JC+G+I | 105 | 35838.060 | 34868.275 | -17328.991 | 0.00 | 0.30 | 0.50 | 0.250 | 0.250 | 0.250 | 0.250 | 0.083 | 0.083 | 0.083 | 0.083 | 0.083 | 0.083 | 0.083 | 0.083 | 0.083 | 0.083 | 0.083 | 0.083 |
| K2+I | 105 | 37020.380 | 36050.594 | -17920.150 | 0.35 | n/a | 0.81 | 0.250 | 0.250 | 0.250 | 0.250 | 0.069 | 0.069 | 0.112 | 0.069 | 0.112 | 0.069 | 0.069 | 0.112 | 0.069 | 0.112 | 0.069 | 0.069 |
| TN93+I | 109 | 37035.602 | 36028.884 | -17905.284 | 0.35 | n/a | 0.81 | 0.250 | 0.208 | 0.229 | 0.313 | 0.057 | 0.063 | 0.114 | 0.069 | 0.127 | 0.086 | 0.069 | 0.116 | 0.086 | 0.091 | 0.057 | 0.063 |
| T92+I | 106 | 37038.302 | 36059.284 | -17923.492 | 0.35 | n/a | 0.81 | 0.229 | 0.229 | 0.271 | 0.271 | 0.063 | 0.075 | 0.122 | 0.063 | 0.122 | 0.075 | 0.063 | 0.103 | 0.075 | 0.103 | 0.063 | 0.075 |
| GTR+I | 112 | 37038.666 | 36004.249 | -17889.958 | 0.35 | n/a | 0.81 | 0.250 | 0.208 | 0.229 | 0.313 | 0.071 | 0.054 | 0.114 | 0.085 | 0.127 | 0.093 | 0.059 | 0.115 | 0.075 | 0.091 | 0.062 | 0.054 |
| HKY+I | 108 | 37074.405 | 36076.920 | -17930.305 | 0.35 | n/a | 0.81 | 0.250 | 0.208 | 0.229 | 0.313 | 0.058 | 0.064 | 0.139 | 0.069 | 0.102 | 0.087 | 0.069 | 0.092 | 0.087 | 0.111 | 0.058 | 0.064 |
| JC+I | 104 | 37147.557 | 36187.005 | -17989.359 | 0.35 | n/a | 0.50 | 0.250 | 0.250 | 0.250 | 0.250 | 0.083 | 0.083 | 0.083 | 0.083 | 0.083 | 0.083 | 0.083 | 0.083 | 0.083 | 0.083 | 0.083 | 0.083 |
| GTR | 111 | 38787.643 | 37762.459 | -18770.066 | n/a | n/a | 0.77 | 0.250 | 0.208 | 0.229 | 0.313 | 0.072 | 0.055 | 0.107 | 0.086 | 0.128 | 0.096 | 0.060 | 0.116 | 0.076 | 0.086 | 0.064 | 0.055 |
| TN93 | 108 | 38789.755 | 37792.270 | -18787.980 | n/a | n/a | 0.77 | 0.250 | 0.208 | 0.229 | 0.313 | 0.058 | 0.064 | 0.108 | 0.070 | 0.128 | 0.088 | 0.070 | 0.116 | 0.088 | 0.086 | 0.058 | 0.064 |
| K2 | 104 | 38804.614 | 37844.062 | -18817.887 | n/a | n/a | 0.77 | 0.250 | 0.250 | 0.250 | 0.250 | 0.071 | 0.071 | 0.109 | 0.071 | 0.109 | 0.071 | 0.071 | 0.109 | 0.071 | 0.109 | 0.071 | 0.071 |
| T92 | 105 | 38820.600 | 37850.815 | -18820.261 | n/a | n/a | 0.77 | 0.229 | 0.229 | 0.271 | 0.271 | 0.064 | 0.076 | 0.119 | 0.064 | 0.119 | 0.076 | 0.064 | 0.100 | 0.076 | 0.100 | 0.064 | 0.076 |
| HKY | 107 | 38853.478 | 37865.227 | -18825.461 | n/a | n/a | 0.77 | 0.250 | 0.208 | 0.229 | 0.313 | 0.059 | 0.065 | 0.135 | 0.071 | 0.098 | 0.089 | 0.071 | 0.089 | 0.089 | 0.107 | 0.059 | 0.065 |
| JC | 103 | 38921.337 | 37970.019 | -18881.868 | n/a | n/a | 0.50 | 0.250 | 0.250 | 0.250 | 0.250 | 0.083 | 0.083 | 0.083 | 0.083 | 0.083 | 0.083 | 0.083 | 0.083 | 0.083 | 0.083 | 0.083 | 0.083 |

**NOTE**: -- Models with the lowest BIC scores (Bayesian Information Criterion) are considered to describe the substitution pattern the best. For each model, AICc value (Akaike Information Criterion, corrected), Maximum Likelihood value (*lnL*), and the number of parameters (including branch lengths) are also presented [1]. Non-uniformity of evolutionary rates among sites may be modeled by using a discrete Gamma distribution (+*G*) with 5 rate categories and by assuming that a certain fraction of sites are evolutionarily invariable (+*I*). Whenever applicable, estimates of gamma shape parameter and/or the estimated fraction of invariant sites are shown.Assumed or estimated values of transition/transversion bias (*R*) are shown for each model, as well. They are followed by nucleotide frequencies (*f*) and rates of base substitutions (*r*) for each nucleotide pair.Relative values of instantaneous *r* should be considered when evaluating them. For simplicity, sum of *r* values is made equal to 1 for each model. For estimating ML values, a tree topology was automatically computed. This analysis involved 53 nucleotide sequences. Codon positions included were 1st+2nd+3rd+Noncoding. There were a total of 1977 positions in the final dataset. Evolutionary analyses were conducted in MEGA X [2]

*Abbreviations:* TR: General Time Reversible; HKY: Hasegawa-Kishino-Yano; TN93: Tamura-Nei; T92: Tamura 3-parameter; K2: Kimura 2-parameter; JC: Jukes-Cantor./div>

1. Nei M. and Kumar S. (**2000**). *Molecular Evolution and Phylogenetics*. Oxford University Press, New York.

2. Kumar S., Stecher G., Li M., Knyaz C., and Tamura K. (**2018**). MEGA X: Molecular Evolutionary Genetics Analysis across computing platforms. *Molecular Biology and Evolution* **35**:1547-1549.
